# Supplementary material for: A Synthetic CPP33-Conjugated HOXA9 Active Domain Peptide Inhibits Invasion Ability of Non-Small Lung Cancer Cells
Source: Biomolecules. 2020 Nov 23;10(11):1589. doi: 10.3390/biom10111589 (PMC7700116; doi:10.3390/biom10111589)
Supplement: Supplementary file 1 [file biomolecules-10-01589-s001.pdf]

Table S1. Primer sequences for producing partial fragments of HOXA9.

Gene	Primer sequences	Annealing temperature	Size of amplicon (bp)
HOXA9-N motif	Forward: 5′-AAA GAA TTC ATG GCC ACC ACT GGG GCC-3′<br>Reverse: 5′-AAA GCG GCC GC TCA GTC CGG CGC CGC CGC CGC-3′	60°C	335
HOXA9-M motif	Forward: 5′-AAA GAA TTC ATG GTG CAC CCC CAG GCG CCC-3′<br>Reverse: 5′-AAA GCG GCC GC TCA ATT GGG ATC GAT GGG GGG-3′	60°C	335
HOXA9-C motif	Forward: 5′-AAA GAA TTC ATG AAT GAG AGC GGC GGA GAC-3′<br>Reverse: 5′-AAA GCG GCC GC TCA CTC GTC TTT TGC TCG GTC-3′	60°C	289

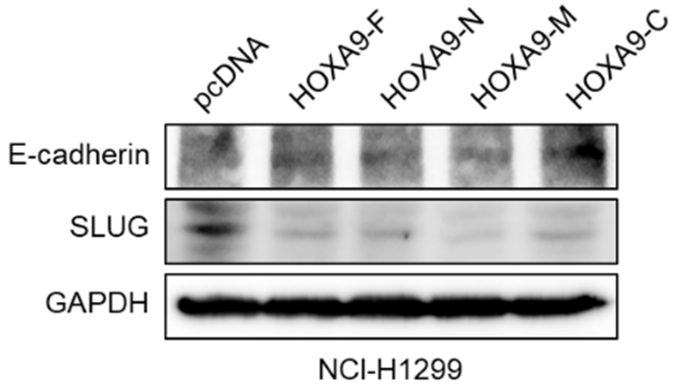

Figure S1. The effect of HOXA9 full-length or partial fragments on change of E-cadherin and SLUG protein expression in NCI-H1299 cells. The expression was analyzed by western blotting.

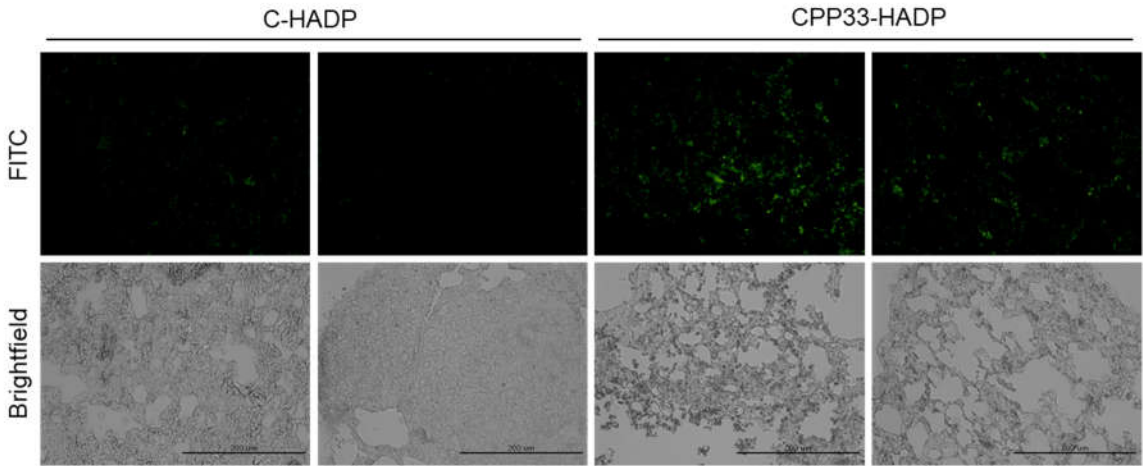

Figure S2. Visualization of cell penetrating peptides using fluorescence microscopy in C-HADP or CPP33-HADP treated xenograft lung tissues. All images are shown at 20X magnification.
